# Production-Level Mitigation of Mn(VII) via a Novel Quaternary Hybrid Nanocomposite: Structural Elucidation, Experimental Optimization, and Advanced Ionic Simulation

**DOI:** 10.3390/nano16120742

**Published:** 2026-06-13

**Authors:** Raouf Hassan, O. A. Mohamed, M. Rashad, Ahmed S. Elshimy

**Affiliations:** 1 Civil Engineering Department, College of Engineering, Imam Mohammad Ibn Saud Islamic University (IMSIU), Riyadh 13318, Saudi Arabia; 2 Environmental Science and Industrial Development Department, Faculty of Postgraduate Studies for Advanced Sciences, Beni-Suef University, Beni-Suef 62511, Egypt; ola.abdelaziz.mohamed@psas.bsu.edu.eg; 3 Physics Department, Faculty of Science, University of Tabuk, Tabuk 71491, Saudi Arabia; m.ahmad@ut.edu.sa; 4 Faculty of Earth Science, Beni-Suef University, Beni-Suef 62511, Egypt; ahmed.salah2407@esc.bsu.edu.eg

**Keywords:** waste valorization, statistical modeling, scale up, advanced ionic theory, experimental analyses

## Abstract

This study was conducted to investigate a novel quaternary hybrid nanocomposite (QHNC) that can successfully remove Mn(VII) ions from contaminated water. The nanocomposite was analyzed using FTIR, XRD, BET, TGA/DTG and FESEM/EDX techniques to investigate whether the synthesis led to an outcome with optimal properties that will enable it to effectively remove Mn ions from aqueous solutions. Optimal results have been achieved by conducting the analysis at a pH level of 2, using 25 mg of the adsorbent material, an interaction time of 60 min and a concentration of 500 mg/L. The Freundlich isotherm best described the adsorption equilibrium. Further analysis through advanced computational simulations indicated that a sorption process underpins the phenomenon based upon a complex geometry mechanism with a preferential horizontal to inclined orientation of the adsorbate upon the surface. The techno-economic assessment reveals the biosorbent’s viability—with a production cost that is highly competitive at USD 9.95/kg, yet with a stable removal efficiency of nearly 60% over five cycles. Such factors lead to a treatment cost of around USD 7.3 for 1 m^3^ of 500 mg/L Mn(VII)—confirming both the economic viability and scalability for advanced tertiary wastewater remediation applications.

## 1. Introduction

Contamination of the environment creates a serious danger to the global balance, adversely affecting the health of humans, biodiversity, the ecosystem, and global balance. Research has established that living in an area where there is contamination results in deaths from diseases of the heart and lungs; further, toxic substances that cause nervous system harm lead to brain malformation in children [1]. The serious problem of water pollution comes from several sources of human activities such as industrial waste [2], agricultural runoff [3], untreated effluent discharge [4], and urban expansion discharge [5]. Indeed, manganese in the heptavalent state (Mn(VII)), as potassium permanganate (KMnO_4_), represents a substantial environmental and toxicological threat. Manganese introduction in hydrologic systems mainly stems from various forms of industrial processes, which include ore extraction, refining operations, iron and steel production, and the burning of coal [6,7,8]. The key element responsible for water quality deterioration in terms of unfavorable organoleptic characteristics, such as changes in taste, color, and turbidity, resulting in adverse economic and sanitary implications, is manganese ions [7]. The long-term use of water containing higher levels of manganese content than the recommended levels results in an increased probability of carcinogenesis and the development of health-related problems in the respiratory system, liver, and neurological system [8]. As a measure aimed at protecting people from these dangers, the World Health Organization (WHO) has issued a guideline value based on health criteria that restricts the manganese content in drinking water to up to 0.1 mg/L [9]. Hence, it is essential to develop methods capable of treating contaminated water sources to protect people from these consequences.

Numerous studies have proven that a variety of techniques, including but not limited to chemical oxidation, ion exchange, coagulation/flocculation, degradation, ultrafiltration, electro-adsorption, and ozonation, have been applied successfully in removing contaminants from water [10,11]. Nevertheless, there are serious limitations associated with some of these techniques, including harmful sludge formation, inefficiency in the elimination of contaminants, a long duration required for treatment, a high consumption of energy, and high costs involved. In contrast, adsorption can be considered an effective separation technique, with its effectiveness being manifested through its ease of use, efficiency, non-toxic nature, and low cost, resulting in strong support for its application to control water pollution. Some of the adsorbents used in adsorption include but are not limited to multi-walled carbon nanotubes (MWCNTs) [12], carboxylic acid functionalized MWCNTs [12], iron-fortified pumice [13], marine nanosediments [14], microwave-activated bentonite [15], and chemically modified anthracite [16]. Among the newest directions in this area, one may name the recycling of industrial waste products enriched with minerals, as well as agricultural residues, into reusable absorbents, which will allow for the removal of Mn(VII) species from water solutions [17,18]. Kaolin is known to be an interesting raw material due to its natural plasticity. Its structure includes a silica tetrahedron and alumina octahedron layer, and its theoretical composition includes 46.54% SiO_2_, 39.5% Al_2_O_3_, and 13.96% H_2_O [19]. One of the main practical applications of kaolin involves the production of alum, which is a widely used coagulant and flocculant, which means that it is made of aluminum salts used at water purification plants [20]. The wide application of kaolin leads to a number of problems, as improper usage causes both environmental issues and economic problems. Thus, there appears to be a great amount of kaolin industrial waste product called dealuminated kaolin (DK).

Noteworthy in this context is the formation of eco-friendly biochar adsorbents from agro-waste materials based on their worldwide accessibility, local availability, and minimal requirement for pre-treatment, factors that make them a sustainable material [21,22,23]. Academic studies have reported the production of biochars from a wide variety of wastes. Such residues include fruit waste remains such as apricot stone [24] and plum stones [25], rice husk [26], hazelnut shell [27], and pine cone [28]. Additionally, there have been several studies on the adsorption capabilities of materials derived from coconut shells. These include coir pith [29], husk [30], and shell [31]. In a study by Patra et al. [32], it has been reported that biochar obtained from agricultural waste has a high surface area, functional groups, and surface charges, making it a powerful adsorbent for heavy metals [33]. Prior studies using untreated agricultural waste adsorbents in removing mercury [33] and lead [34] also showed promising results.

Identified as an eco-friendly and cost-effective material, chitosan (CHO) represents a promising material due to its renewability and low level of toxicity. The high level of biocompatibility and environmental compatibility of CHO are recognized, which make this compound a promising one for use in composite structures. In addition to carbonaceous matrices, CHO can be used to develop adsorbents with improved performance, namely, those that exhibit enhanced contaminant adsorption capacities. However, even though activated carbon and CHO are often used as adsorbents to reduce pollution levels, both of them have certain drawbacks, including poor efficiency, instability, sensitivity to acids, and inability to adsorb some waterborne heavy metal ions. It is suggested that developing a ternary nanocomposite containing all these components can overcome these problems and ensure the development of a more effective and cost-effective solution. At the same time, titanium dioxide is another component with several additional benefits, such as nontoxicity, high chemical stability, biocompatibility, and ability to interact with organic compounds [35]. Thus, using CHO in a dealuminated kaolin waste (DKW)/activated carbon (AC)/TiO_2_ nanocomposite will produce a multifunctional adsorbent for pollutant reduction.

For the present investigation, a new biosorbent referred to as QHNC has been developed. This is the first use of this waste material in this combination, which provides an extremely high Mn adsorption capacity with up to 338.75 mg/g. Comprehensive characterization of the material through spectroscopy (FTIR, XRD, TGA/DTG), surface analysis (BET surface area, FESEM/EDX and elemental mapping) demonstrated its efficiency, making it an important advancement in wastewater purification technology. The adsorption kinetics and equilibrium were estimated based on well-established theoretical approaches. In order to study specific reactions occurring between Mn(VII) ions and the active sites of the QHNC biosorbent, advanced theoretical models were employed. Thus, a novel adsorbent obtained from biopolymers, solid biogenics, inorganic waste, and metallic nanoparticles has been successfully designed in the current investigation. Moreover, the interfacial chemical mechanisms responsible for the Mn(VII) sorption process have been thoroughly analyzed.

## 2. Materials and Methods

### 2.1. Materials and Chemicals

The dealuminated kaolin waste (DKW) was sourced from Egyptian Alum Factory located in Qalyubia City, Egypt. On the other hand, activated carbon (AC) obtained from plant waste used in the experiment came from Nansha District, Guangzhou, Guangdong Province, China. In this regard, the concentrated synthetic solution containing heavy metals was prepared using potassium permanganate (KMnO_4_; molecular weight = 319.85 g/mol), which is a premium quality metallic salt supplied by Sigma-Aldrich (Malaysia). Hydrochloric acid and other analytical reagents were purchased from Sigma-Aldrich Co. (St. Louis, MO, USA), while ammonium was obtained from Research Lab Fine Chem Industries Ltd., Mumbai, India. The CHO and titanium nanoparticles (TiO_2_) were supplied from Alpha Chemica, Mumbai, Maharashtra (India).

### 2.2. Fabrication of QHNC Adsorbent

**Figure 1:**
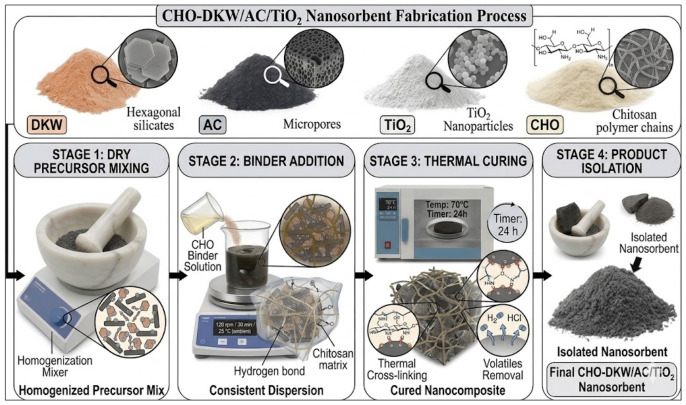
Flowchart explaining the fabrication process for the QHNC nanosorbent.

The preparation of the QHNC nanosorbent involved an overall synthesis process represented by Figure 1. Initially, a dry mixture of all the solid precursors was prepared whereby 2.5 g of DKW, 2.5 g of AC, and 0.5 g of TiO_2_ nanoparticles were combined. The dry mixture was introduced into 20 mL of the CHO binder solution. The CHO solution consisted of 1 g of CHO dissolved in 100 mL of 1% (*v*/*v*) aqueous hydrochloric solution. In the acidic environment, the CHO is soluble, and hence, a gel matrix can form. This resulted in the formation of a composite that was stirred at 120 rpm for 30 min at room temperature. Stirring ensures an adequate distribution of the solid constituents within the CHO matrix and embedding of the TiO_2_ nanoparticles into the structure. After stirring, the nanomaterial was thermally treated at 70 °C for 24 h. Thermal treatment of the precursor plays two major roles. It aids in the evaporation of the solvent while, at the same time, cross-linking the CHO to provide a solid structure containing AC, DKW, and TiO_2_.

### 2.3. Characterization of the Created QHNC Biocomposite

The mineralogical characterization of both the raw DKW and AC materials as well as the QHNC composite material biosorbents was carried out using X-ray diffraction (XRD). The XRD experiments for all materials were conducted using the PANalytical Empyrean diffractometer instrument with a copper Kα X-ray source (wavelength of 0.154 nm). Diffraction patterns were collected using a 2θ angular range of 5–70°, with a tube voltage and current of 40 kV and 30 mA, respectively. For identifying the surface functional groups in all materials, FTIR analysis was carried out for the raw materials of DKW, AC, and the final nanocomposite. FTIR analysis was carried out for all the materials using a Bruker VERTEX 70 spectrometer, covering the spectral range from 390 cm^−1^ to 4000 cm^−1^. In addition, the thermal behavior and decomposition of the QHNC material were assessed using thermogravimetric analysis (TGA). The thermal decomposition and weight loss behavior of the samples were determined using TGA-DTG. These tests were conducted by means of a thermal analyzer from Stream Instrumentation (France) in which the materials were subjected to heating in a nitrogen environment with a constant flow of 50 mL/min to reach temperatures ranging between ambient condition (30 °C) and 950 °C at a rate of 10 °C per min. Morphology investigation of the QHNC biocomposites was performed using a Thermo Fisher Scientific Quanta FEG 250 field emission scanning electron microscope (FESEM), which is manufactured by Thermo Fisher Scientific (originally FEI) and sourced from Hillsboro, OR, the United States. Moreover, simultaneous analysis by energy-dispersive X-ray spectroscopy (EDX) enabled the establishment of the presence and distribution of elements within the samples. Properties such as porosity and surface area of the biocomposites were investigated through nitrogen adsorption isotherm testing, measured using a Micromeritics TriStar II 3020 apparatus (which is headquartered in Norcross, Georgia, United States) at 77 K. Data obtained from the adsorption/desorption isotherms were analyzed via Brunauer–Emmett–Teller (BET) theory.

### 2.4. Mn(VII) Adsorption Experiments

The efficiency of the nanocomposite as a removal agent of Mn(VII) ions from aqueous media was investigated using batch equilibrium experiments. This investigation included the analysis of the operational parameters, such as the pH (2–9), dosage of the adsorbent (5–40 mg), duration of interaction between the aqueous solution and adsorbent (5–120 min), and the initial concentration of metal ions (50–600 mg/L). Experiments were performed in conical test tubes containing 50 mL of aqueous media. Investigation of the adsorption isotherms included immersion of the adsorbent in an aqueous Mn(VII) solution with concentrations ranging from 50 to 600 mg/L at 25 °C. Kinetic experiments involved using an aqueous medium with a concentration of Mn(VII) ions of 500 mg/L; the medium was sampled at certain time points between 5 and 120 min of interaction with the adsorbent. All the experiments were performed using 25 mL of aqueous solution of Mn(VII) ions and stirring the medium for 120 min at a speed of 200 rpm. Centrifugation and quantification of the residual Mn(VII) ions in the solution were performed using the spectrophotometric method. At low pH (e.g., pH = 2), Mn(VII) is reduced to colorless Mn(II), requiring correction. Mn(II) was measured colorimetrically via persulfate oxidation with Ag^+^ as a catalyst: the sample was treated with AgNO_3_ and (NH_4_)_2_S_2_O_8_ at room temperature to form purple MnO_4_^−^, and the absorbance was read at 525 nm. The calculation of Mn(VII) adsorption was carried out by Equations (1) and (2).

(1) $$q_{e}=(C_{O}-C_{e})\frac{V}{m}$$

(2) $$q_{t}=(C_{O}-C_{t})\frac{V}{m}$$

The fundamental parameters in this study are defined as follows: *C_O_* denotes the initial Mn(VII) concentration (mg/L), *C_e_* is its equilibrium concentration, and *C_t_* is the residual concentration at time *t*. *V* signifies the solution volume in liters, and *m* represents the mass in grams of the QHNC biosorbent.

### 2.5. Modeling of Mn(VII) Adsorption on QHNC

A detailed analysis of the physical and chemical characteristics of the equilibrium models is required for a proper understanding of the laws of the sorption process of Mn(VII) ions from water solutions. Scientists have traditionally been using Langmuir and Freundlich isotherms, which are two well-known formulas useful for describing adsorption experiments conducted under laboratory conditions [36,37]. Despite the fact that they allow for describing the general pattern of the process, both of these empirical methods are absolutely unable to solve particular microscopic issues. To gain better knowledge of the nature of the interaction between the sorbate and the sorbent, including the binding of a single atom and other interactions of higher magnitude, more sophisticated models based on statistical physics should be applied [38,39]. They will enable the obtainment of such characteristics as the number of binding centers, energy distribution, and other parameters, which could not be identified otherwise. In other words, it is important to combine theoretical knowledge obtained through experimental observations with a series of models varying from classical approaches to atomistic calculations. This makes it possible to find explanations for the adsorption geometry, taking into account its reasons. All protocols relating to the measurements are presented in the Supplementary Materials.

### 2.6. Regeneration and Desorption

The process of material regeneration is vital in the course of carrying out the recycling process, since it ensures that the substance is returned to its original state to allow continued usage. In this research, the adsorbent material used has been successfully regenerated through the process of desorption of Mn(VII) from the material surfaces. The process of desorption was achieved through the use of 50% ethanol. From the adsorption process carried out with the aid of 500 mg/L Mn(VII) solution, 25 mL of the 50% ethanol solution was used and agitated for one hour. The process was conducted in a flask at room temperature and stirred for 60 min. Afterwards, the process of separation through centrifugation was performed, and measurement of the concentration of Mn(VII) in the supernatant was carried out. The adsorbent material was washed with distilled water to regenerate it and check its reusability through several reuse cycles. The process was conducted using Equation (3).

(3) $$R\;\left(\%\right)=\frac{C_{O}-C_{e}}{C_{O}}\cdot 100$$

This successful regeneration highlights how sustainable resource management enables practical recovery and repeated application of functional materials.

## 3. Results and Discussion

### 3.1. Characterization of Created QHNC Biosorbent

#### 3.1.1. XRD Investigations

**Figure 2:**
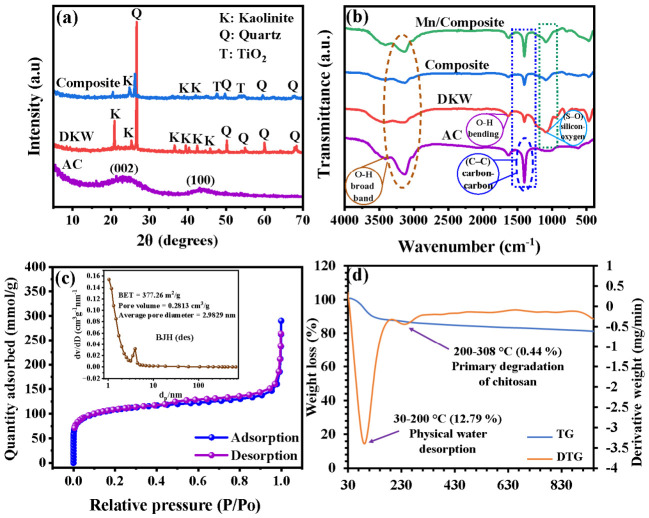
(**a**) XRD and (**b**) FTIR of AC, DKW, and the QHNC biocomposite, and (**c**) BET surface area and (**d**) TG/DTG curves of the QHNC biocomposite.

The XRD patterns of the DKW, AC, and as-synthesized QHNC biocomposite, which show the crystal structures of these materials, are presented in Figure 2a, in the 2θ region of 5–70°. The AC shows two major broad scattering XRD signals, namely, at 23.58° and 43.74° 2θ, respectively. From the comparison with standard diffraction patterns (ICDD 00-041-1487), these peaks are attributed to the (002) and (100) planes, respectively. The wide broadening of the above peaks is characteristic of the carbon matrix with a low degree of long-range ordering. Thus, the presence of an amorphous carbonaceous matrix confirms our previous findings concerning the structure of carbon materials obtained from biomass [40]. Furthermore, the X-ray diffraction (XRD) spectrum of the DKW sample shows three sharp well-resolved peaks located at around 26.78°, 50.14°, and 59.98°, which are probably related to the presence of quartz minerals [41] with ICDD card 00-046-1045. Other peaks have been registered at 20.86°, 25.30°, and 42.46° in the XRD pattern of the DKW samples, which can be associated with the presence of kaolinite [42] with ICDD card number 00-014-0164. The formation of TiO_2_ (ICDD 00-021-1272) after nanocomposite synthesis has been confirmed by XRD analysis, since the characteristic anatase peaks appeared at 47.50° and 53.6°. The inability to crystallize chitosan prevented its detection via XRD in the nanocomposite absorbent [43]. Its presence was hence verified through the application of both FTIR and FESEM analysis methods, whereby the former determined its chemical composition, while the latter indicated its morphology. According to the XRD results, the synthesis of the QHNC biocomposite has been completed successfully. The XRD analysis showed that crystalline compounds are formed in the QHNC biocomposite, including those of quartz, kaolinite present in DKW as the raw material, as well as TiO_2_.

#### 3.1.2. FTIR Spectroscopy

The evolution of functional groups from the precursors to the end composite was characterized using FTIR spectroscopy. The FTIR spectra of the DKW, AC and QHNC sorbent are shown in Figure 2a. In the case of the AC sample, its FTIR spectrum is comprised of four peaks, which are positioned at 3124 cm^−1^, 1635 cm^−1^, 1400 cm^−1^, and 1024 cm^−1^. The broad peak at ~3124 cm^−1^ is related to the presence of O–H stretching vibrations, which confirms the phenolic hydroxyl groups [44]. In turn, the peak at 1635 cm^−1^ represents C=O stretching vibrations, whereas the peaks at 1400 cm^−1^ and 1024 cm^−1^ can be associated with C–C bending and C–O stretching vibrations, respectively [45]. Concerning the DKW sample, some important vibration frequencies of this material are represented by the peaks at 3446 cm^−1^ (O-H stretching vibrations), 1638 cm^−1^ (H-O-H bending vibrations), and 1095 and 466 cm^−1^ (asymmetric and bending vibrations of the Si-O-Si tetrahedral linkage, respectively) [46]. Moreover, the FTIR band at 465 cm^−1^ of DKW is associated with Si-O stretching vibrations [47]. The signal detected at 797 cm^−1^ corresponds to the bending vibration of the Al–O bond. From the results obtained from QHNC analysis through FTIR, the formation of the nanocomposite was successful. The evidence provided is based on the changes witnessed in the vibration process, such as the blending of certain bands and a decrease in their intensity. The change is probably attributed to the development of microcrystalline chitosan, resulting from the use of an ionic liquid [48] for the preparation of chitosan, thus enabling its dissolving and restructuring. An additional proof that chitosan was incorporated into DKW/AC is the detection of the C–C stretch vibration band at 1095.51 cm^−1^ [49]. Upon manganese loading (Mn/Composite), the structural integrity of the composite was maintained; however, a significant enhancement and sharpening of the peak at ~1400 cm^−1^ and of the O–H stretching region were observed. Moreover, subtle shifts and intensity variations in the Si–O region (~1090 cm^−1^) suggest the successful coordination or attachment of manganese species to the active oxygen-containing functional sites of the composite matrix.

#### 3.1.3. Bet-Derived Surface Analysis

**Figure 3:**
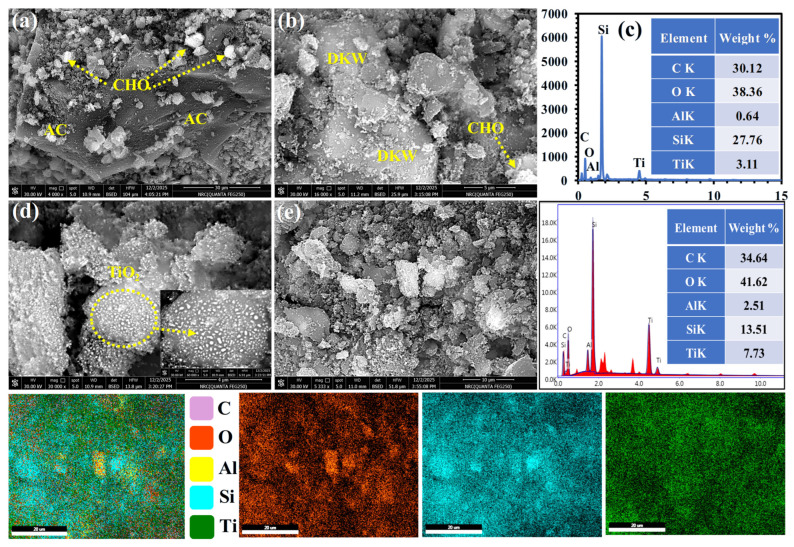
Morphological and elemental characterization of the QHNC biosorbent using FESEM (**a**–**d**) and integrated FESEM/EDX (**e**) with mapping.

The porous system of the QHNC biocomposite is crucial to its function, as indicated by the type of N_2_ adsorption–desorption isotherm with an H3 hysteresis loop (Figure 2c). Through the BET analysis, one obtains information on the specific surface area equal to 377.26 m^2^/g and average pore diameter equal to 2.8929 nm. These results suggest that the structure possesses pores composed mainly of connected mesopores. Such properties ensure that there is a large surface area coupled with the presence of molecular transport channels, providing an ideal medium for species interactions. Therefore, such a pore system enables an optimal environment for the increased kinetics of the sorption and increased final sorption capacity of Mn(VII) ions. The obtained biocomposite has a pore volume of 0.2813 cm^3^/g. In addition to possessing a large specific surface area, it also offers the necessary pore size that allows the optimal diffusion of contaminants, leading to a higher removal of Mn(VII) ions. The biocomposite material has been engineered to be more effective owing to the combined effects of functionality and structure. Apart from having a large specific surface area, it has other features due to its multi-faceted chemistry. In this case, the roles of DKW and AC lie in their use as the main adsorption medium, which features a porous structure containing surface hydroxyl groups. At the same time, the presence of chitosan provides an additional framework, along with introducing a new set of functional groups in the form of amine and hydroxyl groups. There is a direct connection between the roughness of the QHNC surface (Figure 3) and porosity, including the formation of mesoporous and macroporous structures. Such characteristics were confirmed experimentally using the results obtained from the FESEM technique and adsorption studies.

#### 3.1.4. Thermogravimetric Investigations

The thermal stability and decomposition properties of the prepared COS-DKW/AC/TiO_2_ composite were studied by using the TG/DTG analysis technique. From the obtained thermogram shown in Figure 2d, it was found that the compound shows high stability similar to a nanosorbent. From the TGA curve obtained in the range of 30–950 °C, the percentage of total mass loss is quite small (13.23%), leading to an impressive residual mass fraction (86.77%) in the final stage. Analysis of the QHNC nanosorbent by the TG/DTG technique indicates that its thermal process consists of three main phases based on weight loss. The first phase, which takes place in the range of 30–200 °C, involves the removal of moisture by evaporation and desorption processes [50]. The second step occurs from 200 to 400 °C, which can be referred to as the main thermal breakdown stage of the chitosan polymer [45,51], including the dehydration of saccharide rings, depolymerization, and the breakdown of functional groups. It is worth mentioning that the addition of TiO_2_ nanoparticles usually causes a rise in the temperature of the thermal degradation process when compared with pure chitosan, since TiO_2_ acts as a thermal stabilizer. Finally, above 400 °C, the third step consists of the carbonization of organic residues from chitosan and AC. The final weight of the material after the completion of this process denotes the stable inorganic residue composed of TiO_2_, DKW, and the non-combustible parts of AC.

#### 3.1.5. FESEM/EDX and Mapping Interpretations

The study of the elemental composition and morphology of the QHNC nanocomposite was performed by FESEM combined with EDX analysis and elemental mapping. According to the FESEM images (Figure 3), successful synthesis of the heterogeneous four-phase biocomposite used for sorption purposes has been achieved. Analysis of the microstructure reveals considerable morphological diversity that can be explained by a certain structure of AC forming layered flakes, which function as a substrate for the deposition of CHO particulates (Figure 3a). The morphological heterogeneity of the QHNC biocomposite indicates that different formation mechanisms can take place, which is probably related to some local variations in interaction conditions during synthesis. The flake-like shape of the QHNC composite implies a relatively high specific surface area that might be useful for improving the Mn(VII) adsorption effectiveness. The morphology of the heterogeneous biocomposite has been studied in detail in Figure 3b along with the analysis of its elemental composition shown in Figure 3c. The presence of the DKW phase having a stratified architecture associated with the action of the CHO cross-linker was revealed. The QHNC nanosorbent has been shown to contain TiO_2_ nanoparticles in homogeneous distribution throughout the matrix; the particles have an almost spherical shape and tend to separate and to be well-dispersed inside the structure (Figure 3c).

The elemental content of the synthesized biosorbent was analyzed using EDX spectroscopy (Figure 3e). Based on the acquired results, it can be observed that the elemental content consists of silicon (13.51%), titanium (7.73%), and oxygen (41.62%). Moreover, it has been detected that the sample contains alumina at an amount of 2.51% along with the content of carbon (34.64%). Therefore, according to this specific composition, the synthesis of QHNC can be proven, as this particular content proves the complete formation of the biosorbent based on the initial components (i.e., CHO, DKW, AC, and TiO_2_). The distribution of the elements mentioned above was assessed by generating the corresponding distribution map (Figure 3e). As one can see from the maps (in which silicon is marked in light blue, titanium in green, and oxygen in red), there is a good distribution of all the components at the nanoscale level.

### 3.2. Adsorption Investigations of Mn(VII) on QHNC

#### 3.2.1. Effect of Solution pH and QHNC pHPZC on Mn(VII) Uptake

**Figure 4:**
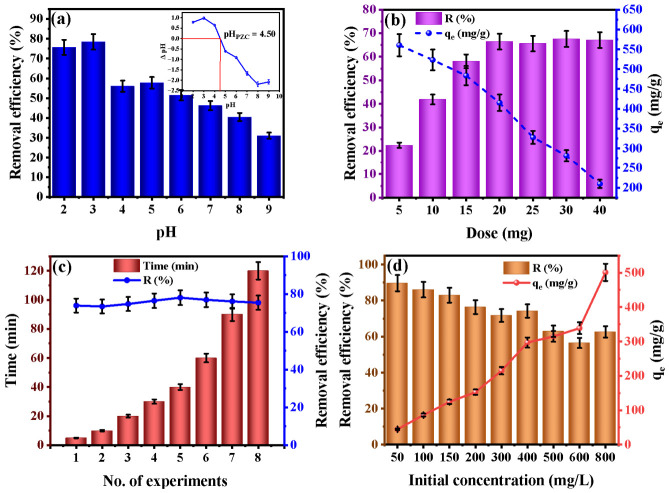
Effects of (**a**) pH, (**b**) adsorbent dose, (**c**) contact time, and (**d**) initial Mn(VII) concentration on the adsorption of Mn(VII) by the QHNC biocomposite.

The influence of solution acidity on the removal efficiency of Mn(VII) ions by QHNC was evaluated in the pH range of 2 to 9. In every test, all factors were kept the same: initial concentration of Mn(VII) ions, 500 mg/L; amount of the adsorbent, 25 mg; temperature, 25 °C; and contact time, 60 min. According to Figure 4a, the pHPZC value of the tested adsorbent was found to be 5.50 [52]. It should be noted that pHPZC means the pH value at which there is no charge on the surface of the adsorbent. Therefore, the efficiency of the adsorption of metal ions will depend significantly on the acidity of the solution due to the negative surface charge of these ions. The adsorption capacity decreases in dependence on the acidity of the solution, starting from 378.3 mg/g at pH = 2 to 155.6 mg/g at pH = 9. The maximum adsorption capacity for Mn(VII) was observed at pH = 2, corresponding to an ion removal efficiency of 75.66%. At a lower solution pH (less than pHPZC—pH < 5.50), a positive surface charge is formed as a result of the protonation of the amine functional groups existing on the surface of the CHO part of the nanocomposite [53]. Due to the positive charge, a high affinity of the biosorbent for the anionic MnO_4_^−^ is achieved. Conversely, at a higher pH compared to pHPZC, there is a negative charge on the surface of the nanocomposite [18], thus causing electrostatic repulsion of the surface from Mn(VII) anions, which significantly affects the efficiency of the process. In other words, the main force involved in the capture of manganese ions by QHNC is electrostatic affinity, which depends greatly on the solution pH.

#### 3.2.2. Influence of QHNC Dosage

Dosage is one of the primary factors responsible for the QHNC biosorbent adsorptive ability towards Mn(VII), as shown in Figure 4b. Such behavior is based on the mechanism associated with changing the number of active adsorption centers. For the study of such a relationship, several experiments with different biosorbent dosages ranging from 5 to 40 mg have been performed. In all of them, the Mn(VII) concentration was maintained constant at 500 mg/L, the temperature was 25 °C, the pH was 2, and the time period was 60 min. The process of Mn species sequestration efficiency depends largely on the dose of the QHNC biocomposite applied. There is a high enhancement in adsorption efficiency from 22.42% to 65.6% when changing the dosage from 5 mg to 25 mg, respectively. A further increase causes minor variations in the removal efficiency (from 65.6% to 67.1% with a 40 mg dosage). This process takes place during two distinct mechanistic stages. The first stage of such progress can be explained by the increasing number of active sites associated with a higher adsorbent dosage. This stage is characterized by a constant plateau that is achieved when the dosage exceeds 30 mg. The plateau is indicative of full saturation of the system in terms of adsorption capacity, due to the fact that the number of manganese ions in the solution significantly exceeds the number of active sites and the low adsorbent efficiency for adsorbing Mn(II). Moreover, this plateau can be viewed as an ideal point, since a complete changeover from the adsorption capacity-limited process to the process controlled by solute concentration occurs. In addition, the adsorption capacity (q_e_) of the nanocomposite towards Mn(VII) is estimated to be 560.5 mg/L at the minimum dose of only 5 mg. Thus, the gradual reduction of q_e_ dependent on the increase in the adsorbent amount demonstrates that there is a non-linear dependence between adsorption capacity and the amount of material used for sequestration.

#### 3.2.3. Impact of Adsorption Time

The interaction adsorption time of Mn ions with the QHNC adsorbent system is one of the most crucial factors of the adsorption kinetics study shown in Figure 4c. Knowing the time required to achieve equilibrium in the adsorption process will help to avoid extra-long time periods during which the effectiveness of the metal extraction would not be higher. In order to research the kinetics of the adsorption process, the adsorption reaction was conducted within the period of 5 to 120 min. For the experiment, a constant amount of the adsorbent of 25 mg, a 500 mg/L concentration of Mn(VII) ions, 25 °C temperature, and pH = 2 were used. The effect of contact time on the removal efficiency was investigated across eight experiments, ranging from 5 to 120 min, as illustrated in Figure 4c. The biosorbent exhibited a remarkably rapid adsorption rate, achieving over 73.9% removal efficiency within the first 5 min. This initial rapid phase is attributed to the abundance of vacant active sites on the adsorbent surface. As the contact time was extended to 120 min, the removal efficiency remained nearly constant, fluctuating within a narrow plateau of approximately 73–78%. This behavior indicates that the system reached equilibrium at a very early stage of the process, at which point the remaining active sites became either saturated or less accessible, or most of them were consumed.

#### 3.2.4. Role of Initial Mn(VII) Concentration

The adsorption capacity of the QHNC biocomposite as an adsorbent was found to be inversely related to the initial Mn(VII) concentration, as illustrated in Figure 4d. The research findings obtained from tests carried out at 50–800 mg/L show that the number of contaminants existing initially will determine their effectiveness in the process [54]. Numerically, the adsorption capacity decreased from 89.6% at the lower level of concentration to 62.6% at the higher level of this range. This inverse relationship can be attributed to changes in the adsorption mechanism. At low Mn concentrations, adsorption will occur due to the availability of numerous adsorption sites. On the contrary, at high concentration levels, the few adsorption sites become saturated, hence leading to competition between different species of Mn. An equilibrium state is thus achieved whereby maximum adsorption capacity is attained by the substance.

### 3.3. Mn Adsorption Kinetic Investigations

**Figure 5:**
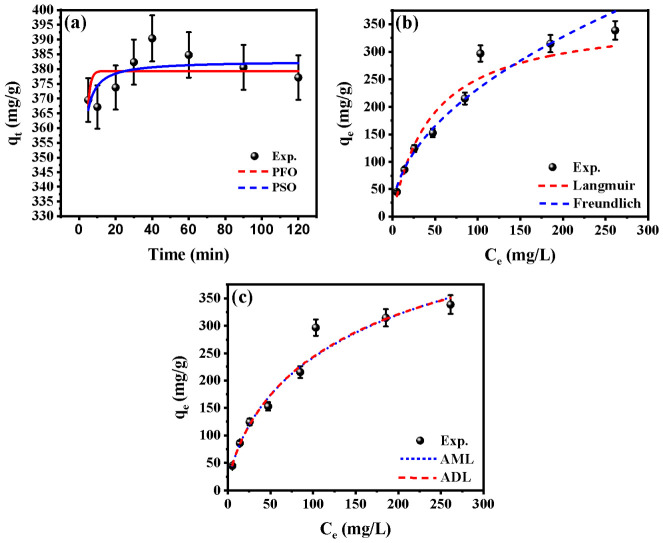
Kinetic modeling and adsorption isotherms of Mn uptake by QHNC nanocomposite: (**a**) nonlinear fitting of PFO and PSO; (**b**) nonlinear Langmuir and Freundlich equations; and (**c**) advanced modeling equations.

The rate-limiting steps and the reaction mechanism of manganese uptake using the QHNC material were identified by means of four well-established kinetic models, namely, Pseudo-First-Order (PFO) and Pseudo-Second-Order models [55,56,57]. These models help us to gain insights into the adsorption process along with providing a basis for understanding the removal process. For fitting the q_t_ (as shown in Figure 5a) values into the PFO and PSO models, nonlinear regression analysis was conducted. It can be seen from Table 1 that the PFO model has much better correlations compared to PSO. This is evident from the fact that the R^2^ of the PFO model is larger than that of the PSO model; the PFO model R^2^ is 0.993, while that of the PSO is 0.646. Furthermore, there is no doubt that the high concordance between the model estimates and the actual dataset results from the small standard deviation of the q_e_ values from the experimental q_t_ values. In other words, this evidence strengthens the applicability of the PFO kinetic model to the Mn(VII) adsorption system. The close agreement in question clearly demonstrates that physisorption is a key process that explains the uptake of Mn(VII) ions by the QHNC nanocomposites. Regarding the kinetic model parameters, the relatively high K_1_ value (0.39 ± 0.1 min^−1^) signifies rapid adsorption kinetics during the initial stage of the process, which is consistent with the sharp increase in the removal efficiency observed within the first few minutes. In contrast, the low K_2_ value (0.002 ± 0.0005 g⋅mg^−1^⋅min^−1^) indicates that, although the initial uptake is fast, a longer contact time is required to transition from the rapid initial phase to the final equilibrium state as the active sites become progressively saturated. Moreover, the FTIR data obtained after Mn(VII) adsorption indicate that a significant enhancement and sharpening of the peak at ~1400 cm^−1^ and of the O–H stretching region were observed. Additionally, subtle shifts and intensity variations in the Si–O region (~1090 cm^−1^) suggest the successful coordination or attachment of manganese species to the active oxygen-containing functional sites of the composite matrix. Therefore, the adsorption process is best described as a complex mechanism involving both physical and chemical interactions.

### 3.4. Isotherm Modeling of Mn(VII) Uptake via QHNC

#### 3.4.1. Fundamental Equilibrium Fitting

Fittings for the Mn(VII) adsorption data obtained experimentally on the QHNC biocomposite using Langmuir and Freundlich isotherm equations have been plotted in Figure 5c, and respective model parameters are presented in Table 2. A comparative analysis has been conducted for two models considering *R*^2^ and *χ*^2^ statistics, which has demonstrated that the fitting based on the Langmuir model [58] is characterized by higher *R*^2^ values and lower *χ*^2^ values. Therefore, it can be argued that the Langmuir isotherm is a better model to describe the Mn(VII) adsorption process. As follows from the experimental data, the mechanism behind the Mn(VII) ion capture by the QHNC biosorbent involves a monolayer adsorption model, which has been confirmed by modeling based on the Langmuir model. According to the results of these calculations, the maximum adsorption capacity (*q_max_*) of the adsorbent equals 365.1 ± 41.9 at 25 °C. Based on the above-mentioned information, the QHNC bio-based adsorbent represents an effective, economical, and eco-friendly option for the purification of Mn(VII)-containing water solutions.

#### 3.4.2. Advanced Statistical Modeling

Simple equilibrium models tend to lack good applicability in the case of explaining the mechanisms of adsorption due to non-corresponding parameters that may not correspond to physical reality [45]. For a complete analysis of the adsorption mechanism involving Mn (VII) and the QHNC biosorbent, there is a need for a more elaborate technique. Such a technique must involve advanced modeling to offer steric and energetic properties at both the macro- and microscopic levels [45]. This is achievable by the application of statistical mechanics models, which consider steric and energetic features. Therefore, these kinds of theoretical approaches provide considerable contributions to the science of water purification by providing a robust framework for understanding empirical data [45]. It can be stated that the adsorption isotherms of Mn(VII) are better fitted by both the advanced monolayer model (AML) and the advanced double layer model (ADL), given their close R^2^ and χ^2^ values. However, among the classical models, the Freundlich model is selected as the best for fitting the isotherm data. In this context, the ADL model was chosen because it accounts for different adsorption layers with varying energies, which supports classical interpretations. In order to better understand the mechanism behind the adsorption process, further calculations were performed related to the determination of the key physicochemical parameters. They involved the number of ions that could be adsorbed on each site (*n*), the density of the monolayer *N_M_*, the theoretically calculated value of the maximum sorption *Q_sat_*, and the energy of the adsorption process Δ*E* [45].

##### Examination of Steric (*n*, *N_M_*, *Nt*, and *Q_sat_*) Parameters

The stoichiometric constant *n* provides information on the configuration of the ions and the interface reaction involved in the QHNC surface. Based on the value of *n*, the nature of the adsorption reaction can be determined. The adsorption reaction involving *n* < 0.5 represents either the shared-site or the horizontal interface mechanism where the adsorbate ion attaches itself to more than one surface site. The adsorption process is heterogeneous when the value of *n* lies between 0.5 and 1.0, showing the existence of different orientations of adsorption. When *n* is greater than 1.0, it shows multi-ion and vertical interface mechanisms where several adsorbate ions are attached to a single surface site. The adsorption of Mn(VII) ions on QHNC shows *n* = 0.6, hence showing the existence of different orientations of adsorption of the Mn species on the biosorbent surface with more attention to horizontal orientation. In addition, the active site concentration (*N_M_*) of the biocomposite is 524.8 mg/g, which indicates the high efficiency of the biocomposite as an adsorbent. The maximum adsorption capacity of Mn(VII), also known as *Q_sat_*, is calculated in the ADL method using the steric parameters (*n* and *N_M_*) by the formula *Q_sat_* = 2*n*·*N_M_* [58,59]. The obtained *Q_sat_* = 591.94 mg/g shows the high efficiency of the biosorbent.

##### Evaluation of Energetic Parameters (Δ*E*)

One important step in the investigation of the Mn(VII)/QHNC interaction is the calculation of the adsorption energy (Δ*E*). This parameter is crucial in finding out which factors prevail in this system [60], and it was estimated following a common procedure [61].

(4) $$C_{1}=C_{s}e^{-\frac{∆E_{1}}{RT}}$$

(5) $$C_{2}=C_{s}e^{-\frac{∆E_{2}}{RT}}$$

Here, *C_s_* represents the solubility of KMnO_4_, while *C*_1_ and *C*_2_ (mg/L) correspond to the adsorbed layers of Mn(VII). *T* is the absolute temperature (K), and *R* denotes the universal gas constant (8.314 J·mol^−1^·K^−1^). The positive value of ΔE suggests a potential endothermic tendency, which was further rigorously confirmed by the comprehensive temperature-dependent thermodynamic analysis detailed in Section 3.5. The obtained ΔE values of 14.4, 15.1, and 15.82 kJ/mol indicate a favorable interaction between Mn(VII) ions and the QHNC surface. The consistently low ΔE values, all below 20 kJ/mol, clearly suggest that physisorption is the dominant mechanism.

### 3.5. Mn(VII) Adsorption Thermodynamics

A linear regression of ln(q_e_/C_e_) against reciprocal temperature (1/T), depicted in Figure S1, was employed to derive the thermodynamic parameters, namely, entropy change (ΔS°), enthalpy change (ΔH°), and Gibbs free energy change (ΔG°), which are compiled in Table S1. The negative magnitudes of ΔG° indicate that Mn(VII) adsorption onto the QHNC surface occurs spontaneously. Moreover, the ΔG° values became increasingly negative as the temperature rose, demonstrating that the spontaneity of the process improves at higher temperatures. Conversely, the positive sign of ΔH° confirms the endothermic nature of the adsorption. Additionally, the positive ΔS° value supports the increased randomness or disorder at the solid–solution interface during adsorption [62].

### 3.6. Probable Mn(VII) Adsorption Mechanism

The implementation of the QHNC biocomposite as a high-performance biosorbent for Mn(VII) removal demonstrates an outstanding electrostatic affinity that is strictly governed by the solution pH, reaching its maximum efficiency of 75.66% at pH = 2. This remarkable pH-dependent sequestration is mathematically validated through advanced statistical mechanics modeling, in which the ADL model successfully outperforms classical paradigms and reveals a steric parameter of *n* = 0.564. This stoichiometric constant directly uncovers a multi-orientational, predominantly horizontal interfacial mechanism, wherein the contaminant species become elegantly anchored across the composite’s active sites. With an exceptional *Q_sat_* of 591.9 mg/g and a high functional site density (*N_M_* = 524.8 mg/g), QHNC proves itself not merely as a conventional adsorbent but as a highly efficient, tailored matrix capable of competing with premier materials in environmental remediation.

A dynamic investigation of the system using nonlinear regression reveals an exceptional fit with the PFO kinetic model, capturing a rapid initial uptake rate that removes over 73.9% of the heavy metal within the first five minutes. Crucially, based on the interpreted classical and advanced physical models, supported by robust FTIR data, the successful attachment of manganese species is validated by the sharpening of the peak at approximately 1400 cm^−1^, along with subtle shifts in the Si–O (around 1090 cm^−1^) and O–H stretching regions, indicating a complex cooperative network of physical and chemical interactions. Energetically, the calculated adsorption energies derived from advanced physical modeling (Δ*E*_1_ = 15.1 kJ/mol and Δ*E*_2_ = 15.28 kJ/mol) remain well below the 20 kJ/mol threshold, explicitly confirming that physisorption is the dominant governing mechanism. A cohesive thermodynamic analysis further substantiates this behavior, as the negative Gibbs free energy and positive enthalpy change reveal a highly spontaneous, endothermically driven process that becomes increasingly thermodynamically favorable at elevated temperatures.

### 3.7. Performance Benchmarking with Conventional Adsorbents

A systematic benchmarking analysis of QHNC in comparison to reference adsorbent materials is provided in Table 3. The assessment process is based on primary parameters that include the solution pH, time of contact, adsorbent mass, operational temperature, number of regeneration processes, and maximum contaminant loading. The adsorbent in question showed a remarkable maximum sorption capacity for Mn(VII) ions equal to 338.8 mg/g. While certain modified materials such as modified anthracite (MAn) exhibit a higher capacity (555.5 mg/g), it is worth noting that QHNC achieves its high performance at a lower operational temperature (25 °C) and demonstrates outstanding durability over five regeneration cycles, outperforming all other listed materials in terms of reusability. This exceptional stability and good capacity under mild temperature conditions strongly underline the practical usability and economic viability of the QHNC nanocomposite compared to most contemporary materials.

### 3.8. Evaluation of Competing Substances on Adsorption Efficacy

**Figure 6:**
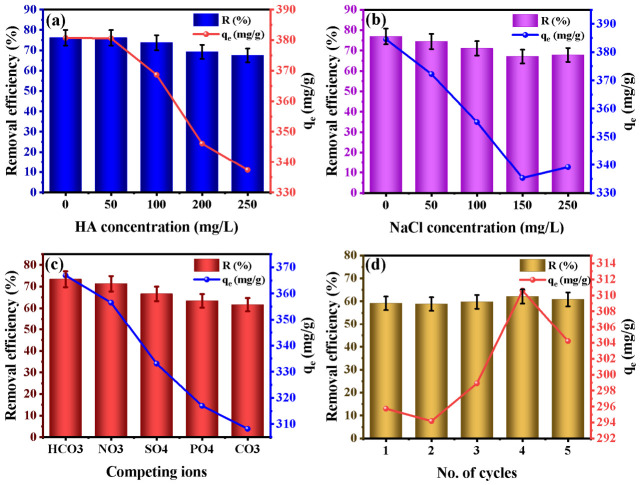
Adsorption performance of the QHNC under varied conditions: (**a**) humic acid concentration, (**b**) ionic strength, (**c**) presence of coexisting ions, and (**d**) regeneration efficiency over multiple cycles.

Competing substances were tested using one of the most common environmental water matrix components, namely NOM. In this case, humic acid (HA) was used in a concentration range of 0–250 mg/L, which allowed assessing whether the adsorbent was capable of occupying competing sites on QHNC. Notably, QHNC proved effective in capturing Mn(VII) ions with efficiencies exceeding 67.5% despite the addition of HA at the maximum possible concentration, as demonstrated in Figure 6a. The decrease in q_e_ with increasing HA concentrations is due to the occupancy of the active sites, decreasing the Mn removal efficiency.

The analysis of the ionic strength reveals the vital importance of this parameter. The increase in the NaCl concentration led to the only decrease in the *q_e_* value from 384.56 to 339.15 mg/g when its concentration was increased from 0 to 250 mg/L (Figure 6b). Such a result indicates that the inhibition effect caused by ionic strength can be explained by the charge screening. To fairly compare the interference of different anions, experiments were conducted at equal anion molar concentration (1 mM) rather than equal mass concentration. This approach eliminates artifacts arising from differences in the molar mass and dissociation stoichiometry (e.g., Na_2_CO_3_ and Na_2_SO_4_ produce twice the sodium ions compared to NaCl at the same mass). The results show that the highest inhibition effect is observed in the case of using CO_3_^2−^ (Figure 6c). The cause of such an effect can be explained by two facts. On the one hand, the high charge density of divalent carbonate ions allows it to effectively compete with permanganate ions for adsorption sites with positive charges [45]. Another reason consists of the ability of carbonate ions to form hydronium ions as a product of their hydrolysis, which lead to the increase in the pH of the solution [45].

### 3.9. Reusability and Regeneration of QHNC

Moreover, the QHNC demonstrates good recyclability, which means that the material can be recycled several times. Within the framework of the specified experimental conditions (initial concentration—500 mg/L, mass of the adsorbent—50 mg, temperature—25 °C, contact time—1 h, pH—2), the studied material shows good prospects for utilization due to economic advantages and environmental friendliness. According to the obtained results (see Figure 6d), 50% ethanol can be successfully utilized as a solution for regeneration. As shown in Figure 6d, the reusability and regeneration of the biosorbent were investigated over five consecutive cycles using the obtained experimental data. The removal efficiency remained relatively stable at approximately 60%. However, a distinct fluctuation in the adsorption capacity (*qe*) was observed, notably peaking at the 4th cycle (311 mg/g) before declining. This behavior is directly attributable to the redox mechanism of Mn(VII). Over successive cycles, the active reducing sites on the biosorbent surface undergo progressive exhaustion and deactivation, leading to a gradual decline in the material’s ability to convert Mn(VII) to Mn(II). This explains the subsequent increase in the residual Mn(VII) concentration and the corresponding decrease in Mn(II). It is possible that the process of cyclic adsorption and desorption of the metal ions leads to changes in the physicochemical properties of the composite material. Such processes might involve pore collapse, changes in the morphology of the surface, or hydrolysis of the polymeric material [45]. All these factors would result in reduced structural integrity, among others, and hence the drop in performance [45].

### 3.10. Approximate QHNC Cost Analysis

From the laboratory synthesis to industrial application of QHNC for water remediation, the economic feasibility of this innovation will determine its success. In the comprehensive fiscal analysis that takes into consideration all costs including those incurred in purchasing precursors, energy used, and production processes, the baseline production price stands at $9.95/kg (Table 4). The economic edge comes with its reusability characteristic since the material is reusable up to at least eight times. Consequently, with the cost of operation per cycle reduced to $1.99/kg while maintaining a removal rate of nearly 60%, the performance versus the cost ratio of QHNC makes it economically attractive. The economics here is based on finding a delicate balance between the adsorption efficacy, which is reflected in the 60% removal rate, and financial constraints [45]. On the other hand, the economics of purchasing raw materials forms another cost factor. Therefore, the QHNC becomes a financially feasible and environmentally friendly method of removing Mn(VII) from water systems. The prices of raw materials and chemicals were largely considered using local prices.

### 3.11. Design and Implementation of Industrial-Scale Performance

Historically, the design of industrial adsorption processes has been based on empirically derived data collected through batch studies conducted in the laboratory. Although continuous flow experiments can be considered more directly applicable to full-scale operations, correlations developed through batch studies have proved sufficiently valid for use in initial design efforts [63]. This project involved developing a prediction equation to describe the scale-up process for Mn(VII) adsorption for the QHNC using batch experimental data. This relationship is presented in Equation (6) [45].

(6) $$\frac{m}{V}=\frac{\left(C_{0}-C_{e})(1+K_{L}C_{e}\right)}{C_{e}q_{max}\;K_{L}}$$

Based on the Langmuir Isotherm constants [64], the scaling approach uses the given volume of Mn(VII) solution (*V*, L) to determine the necessary amount of the QHNC (*m*, g). It takes into account the values of the starting concentration of the solute (*C*_0_) and its concentration at equilibrium (*C_e_*), using the affinity constant (*K_L_*) to express the level of adsorption and *qₘₐₓ* for the saturation point.

**Figure 7:**
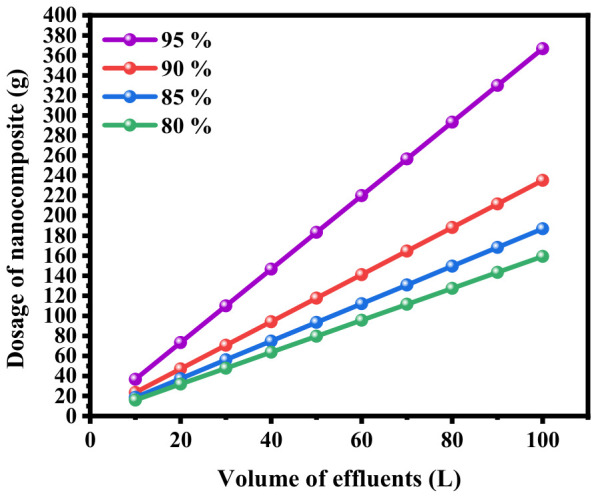
Required quantity of QHNC as a function of target Mn(VII) adsorption efficiency (80–95%) in polluted solutions.

The calculation of the scaling analysis gave the total weight of composite material for high Mn(VII) removal efficiencies of 85–95% for different volumes ranging from 5.0 to 100.0 L based on Equation (6) with constant parameters (C_0_ = 500 mg/L, 25 °C, pH 2.0, 60 min). The economic evaluation becomes justified by the stability of the material throughout five adsorption and desorption processes, which reduces the cost of adsorbent usage to about $1.99/kg per cycle. In order to remove 95% of Mn(VII) ions within a 100 L tank, 366.68 g of QHNC need to be used. However, the usage of this material in cycles reduces the price to $0.73 per 100 L with a removal efficiency of 60%. These values were obtained based on the optimized dosage according to the optimum conditions of the adsorption process in terms of concentration, temperature and time parameters [63]. Equation (6) determines the linear relation of the total amount of composite to the efficiency of the target process in 100 L of a liquid (C_0_ = 500 mg/L). As seen in Figure 7, removal efficiencies of 80%, 85%, 90%, and 95% require the usage of 159.37 g, 186.97 g, 235.32 g, and 366.68 g of adsorbent, respectively.

## 4. Conclusions

A novel sustainable biocomposite, designated QHNC, was synthesized, functionalized, and comprehensively characterized to demonstrate its high efficacy in extracting Mn(VII) ions from aqueous media. Optimal experimental conditions were achieved at pH 2, employing 25 mg of the adsorbent, a contact time of 60 min, and an initial Mn(VII) concentration of 500 mg/L. The equilibrium adsorption data were well described by the Freundlich isotherm model, while the adsorption kinetics followed the pseudo-first-order model. Interpretation based on statistical physics models suggests that each adsorption site accommodates fewer than one Mn(VII) ion, implying a complex adsorption mechanism wherein the adsorbate adopts a predominantly horizontal to inclined orientation. Endothermic adsorption was confirmed by energetic calculations, which further indicated adsorption energies below 20 kJ/mol. A techno-economic assessment underscores the biosorbent’s viability, with a highly competitive production cost of USD 9.95/kg and a stable removal efficiency of nearly 60% maintained over five cycles. Consequently, the estimated treatment cost amounts to approximately USD 7.3 per cubic meter of 500 mg/L Mn(VII)-contaminated water, affirming both the economic feasibility and scalability for advanced tertiary wastewater remediation applications.

## Figures and Tables

**Table 1:** Fitted parameters of adsorption kinetic models for Mn(VII) on QHNC.

Model	Variable	Unit	Value	Std. Error	t-Value	Pr (>|t|)
PFO	*q_e_*	mg/g·min^−1^	379.36 ± 2.98	2.89	131.42	1.31 × 10^−11^
	*K*_1_	min^−1^	0.6 ± 0.2	0.2	3.5	0.01
	*R* ^2^		0.99			
	*X* ^2^		0.99			
PSO	*q_e_*	mg/g	389.76 ± 2.97	2.97	128.59	1.5 × 10^−11^
	*K*_2_	g.mg^−1^·min^−1^	0.01 ± 0.005	0.005	2.29	0.06
	*R* ^2^		0.65			
	*X* ^2^		0.49			

**Table 2:** Estimated parameters of classical and advanced isotherm models for Mn(VII) uptake onto QHNC.

Isotherm Model	Parameters	Values
**Langmuir**	$q_{\max}(mg/g)$	365.1 ± 41.9
	$K_{L}(L/mg)$	0.02 ± 0.005
	*R* ^2^	0.96
	$\chi^{2}$	8.13
**Freundlich**	$K_{F}((mg/g){\left(mg/L\right)}^{-1/nf})$	23.96 ± 4.09
	1/n *f*	0.49 ± 0.04
	*R* ^2^	0.99
	$\chi^{2}$	4.2
**AML**	*n*	0.7 ± 0.16
	*N_M_* (mg/g)	959.9 ± 719.2
	*Q_sat_* (mg/g)	669.8
	∆*E*_1_ (kJ/mol)	14.42
	*R* ^2^	0.99
	$\chi^{2}$	3.6
**ADL**	*n*	0.56
	*N_M_* (mg/g)	524.8
	*Q_sat_* (mg/g)	591.9
	∆*E*_1_ (kJ/mol)	15.1
	∆*E*_2_ (kJ/mol)	15.28
	*R* ^2^	0.99
	$\chi^{2}$	4.35

**Table 3:** Relative Mn(VII) adsorption capacities of reference materials.

Material	pH	Time (min)	Dose (mg)	Cycle Number	Temperature	Q (mg/g)	Ref.
MWCNTs-OCH_2_CO_2_H	6.0	60	30	6	50 °C	238	[12]
MWCNTs	6.0	60	30	6	50 °C	192	[12]
Marine nanosediment	3.0	30	20	-	25 °C	49.2	[14]
Iron impregnated pumice	7.0	1440	500	-	25 °C	7.14	[13]
Modified anthracite (MAn)	3.0	60	30	-	25 °C	555.5	[16]
CHS-DFCW/AC	3.0	60	20	5	55 °C	422.4	[45]
Microwave-treated bentonite	3.0	120	50	4	25 °C	3.17	[15]
QHNC	2.0	60	25	8	25 °C	338.8	This work

**Table 4:** Cost assessment of the QHNC biosorbent.

Material	Experimental Mass Yield (kg/g)	Total Procurement Cost (USD)	Cost per Acquired Unit (kg or g/USD)	Quantity of Material Utilized (by Mass or Volume)	Production Process Material Outlay (USD)
DKW	5 kg	-	-	1 kg	-
AC	4 kg	6.52	1.63	1 kg	0.815
TiO_2_	500 g	36	0.072	200 g	14.4
CHO	150 g	1.5	0.01	80 g	0.8
Production Machinery	Time (h)	Certified power output limit (kW/h)	Energy unit cost (USD/kW·h^−1^)	Total cost	
Dryer	24	1	0.24	5.76	
Stirrer	0.5	1	0.24	0.12	
		Total yield cost = 21.895 USD For 2.2 kg	Total yield cost 9.95 USD/kg		

## Data Availability

The original contributions presented in this study are included in the article/Supplementary Material. Further inquiries can be directed to the corresponding author.

## Supplementary Materials

The following supporting information can be downloaded at: https://www.mdpi.com/article/10.3390/nano16120742/s1, Figure S1. Linear graph of 1/T as a function of ln(q_e_/C_e_) for evaluating thermodynamic parameters. Table S1. Thermodynamics parameters for the adsorption of Mn(VII) on QHNC surface.
