# Supplementary material for: Production-Level Mitigation of Mn(VII) via a Novel Quaternary Hybrid Nanocomposite: Structural Elucidation, Experimental Optimization, and Advanced Ionic Simulation
Source: Nanomaterials (Basel). 2026 Jun 13;16(12):742. doi: 10.3390/nano16120742 (PMC13304526; doi:10.3390/nano16120742)
Supplement: Supplementary file 1 [file nanomaterials-16-00742-s001.zip › nanomaterials-4338567-supplementary.pdf]

Production-Level Mitigation of Mn(VII) via a Novel Quaternary Hybrid Nanocomposite: Structural Elucidation, Experimental Optimization, and Advanced Ionic Simulation

Supplementary Material

2.5.1. Kinetic modeling of Mn(VII) adsorption onto QHNC

A suite of four principal kinetic models was employed to investigate the temporal dynamics and controlling mechanisms of the adsorption process: the pseudo-first-order (PFO) model [1], the pseudo-second-order (PSO) model [2], the intra-particle diffusion (IPD) model [3], and the Elovich kinetic equation [4]. Their foundational mathematical expressions are comprehensively detailed in supporting Eqs S1 through S4.

$$q_t = q_e\ e^{-k_1 t} \quad \text{S1}$$

$$q_t = \frac{q_e^2\, k_2\, t}{q_e k_2\, t+1} \quad \text{S2}$$

$$q_t = k_p\, t^{1/2} + C \quad \text{S3}$$

$$q_t = \frac{1}{\beta}\, Ln(\alpha\beta t + 1) \quad \text{S4}$$

The PFO model provides a rate constant, k_1, expressed in min^{-1}. Correspondingly, the PSO model yields a rate constant, k_2, with units of $g{\cdot}mg^{-1}{\cdot}min^{-1}$. Application of the IPD model reveals two defining values: k_p ($mg{\cdot}g^{-1}{\cdot}min^{0.5}$), the rate constant for diffusion, and C ($mg{\cdot}g^{-1}$), a parameter correlated to the boundary layer's influence. Finally, the Elovich model employs parameters α ($mg{\cdot}g^{-1}{\cdot}min^{-1}$), reflecting the initial chemisorption rate, and β ($g{\cdot}mg^{-1}$), inversely related to the extent of surface coverage.

2.5.2. Conventional isotherm models of Mn(VII) adsorption onto QHNC

Both non-linear isotherm models proposed by Langmuir [5] and Freundlich [6] are formulated mathematically, as detailed in supplemental Eqs. S5 and S6 respectively.

$$q_e = \frac{q_{\max} K_L C_e}{(1+K_L C_e)} \quad \text{S5}$$

$$q_e = K_F \, C_e^{1/n} \quad \text{S6}$$

The Langmuir isotherm defines q_{max} (mg/g) as the theoretical saturation limit for Mn(VII) uptake, with K_L (L/mg) representing the associated equilibrium binding constant. For the Freundlich model, the empirical parameters K_F (mg/g·(mg/L)$^{-1/n}$) and n characterize the relative adsorption capacity and the heterogeneity of site affinities, respectively. Model fitness was assessed by comparing the coefficients of determination (R^2), Chi-square (χ^2) statistics, and root mean square error (*RMSE*) values derived from Eqs. S7 through S9 [7–9].

$$R^2 = 1 - \frac{\sum \left(q_{e,\text{exp}} - q_{e,\text{cal}}\right)^2}{\sum \left(q_{e,\text{exp}} - q_{e,\text{mean}}\right)^2} \quad \text{S7}$$

$$\chi^2 = \sum \frac{\left(q_{e,\text{exp}} - q_{e,\text{cal}}\right)^2}{q_{e,\text{cal}}} \quad \text{S8}$$

$$RMSE = \sqrt{\frac{\sum_{i=1}^{m} \left(Q_{i\,\text{cal}} - Q_{i\,\text{exp}}\right)^2}{p-1}} \quad \text{S9}$$

The term $q_{e,exp}$ (mg/g) signifies the Mn(VII) adsorption capacity quantified through laboratory measurement, whereas $q_{e,cal}$ (mg/g) represents its counterpart generated by mathematical modeling.

2.5.3. Advanced modeling of Mn(VII) remediation

2.5.3.1. Advanced monolayer (AML) model

This model confirmed that the adsorption of Mn(VII) onto QHNC occurs with uniform adsorption energy, leading to the formation of a monolayer of Mn(VII) ions on the biocomposite surface. The corresponding monolayer adsorption equation is presented in Eq. S10 [10].

$$q_e = \frac{nN_M}{1+\left(\frac{C_{1/2}}{C_e}\right)^n} \qquad \text{S10}$$

The parameter $C_{1/2}$ (mg/L) represents the half-saturation concentration required for the formation of a Mn(VII) monolayer on the QHNC surface. The coefficients n and N_M characterize the ionic occupancy per adsorption site and the surface concentration of reactive centers, respectively.

2.5.3.2. Advanced double-layer (ADL) model

The computational modeling confirms a two-layered adsorption mechanism for Mn(VII) onto the QHNC. Each adsorbed layer possesses a characteristic binding energy, denoted as ΔE_1 for the initial attachment of ions to the composite surface, and ΔE_2 for subsequent interactions between adsorbed ions. The derivation of these parameters follows the methodology outlined in Eq. S11 [10, 11].

$$q_e = nN_M \frac{\left(\frac{C_e}{C_1}\right)^n + 2\left(\frac{C_e}{C_2}\right)^{2n}}{1+\left(\frac{C_e}{C_1}\right)^n + \left(\frac{C_e}{C_2}\right)^{2n}} \qquad \text{S11}$$

where, the concentrations C_1 and C_2 (mg/L) correspond to the two distinct layers of adsorbed Mn(VII) ions. The steric coefficient, n, defines the quantity of ions associated with each available site, whereas the parameter N_M characterizes the total concentration of active binding sites present on the nanoscale sorbent material.

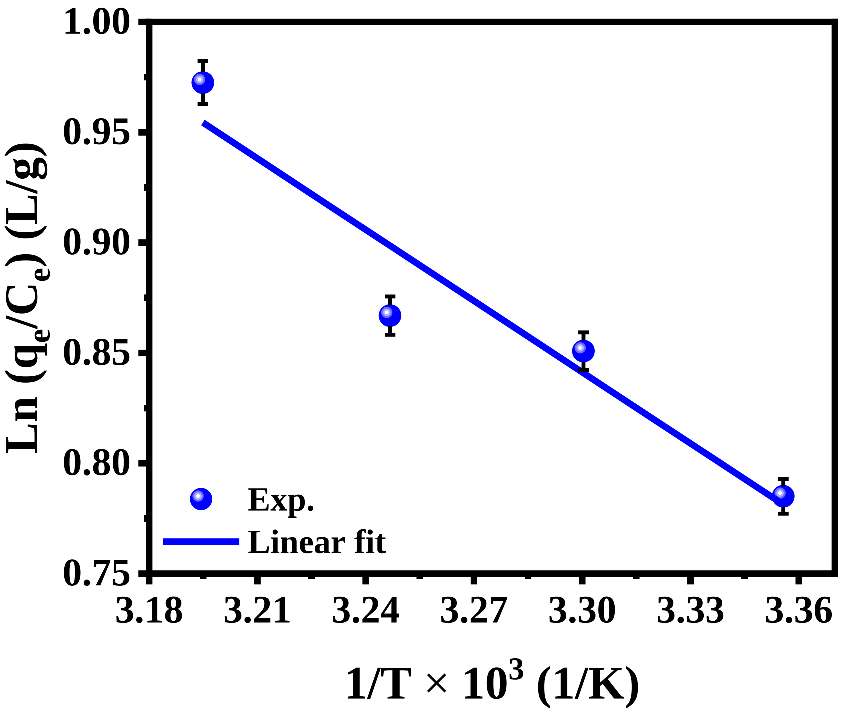

Figure S1. Linear graph of 1/T as a function of ln(q_e/C_e) for evaluating thermodynamic parameters.

Table S1. Thermodynamics parameters for the adsorption of Mn(VII) on QHNC surface.

**Temperature (◦C)**	**ΔG° (kJ/mol)**	**ΔH° (kJ/mol)**	**ΔS° (J/mol K)**
**25**	−1.945	8.95	36.53
35	−2.143		
45	−2.219		
55	−2.531		

References

[1] L. S., ABOUT THE THEORY OF SO-CALLED ADSORPTION OF SOLUBLE SUBSTANCES, 24 (1898) 1–39.

[2] Y.S. Ho, G. McKay, Sorption of dye from aqueous solution by peat, Chemical Engineering Journal 70 (1998) 115–124.

[3] W.J. WeberJr., J.C. Morris, Kinetics of Adsorption on Carbon from Solution, Journal of the Sanitary Engineering Division 89 (1963) 31–59.

[4] M.H. Dehghani, A. Dehghan, A. Najafpoor, Removing Reactive Red 120 and 196 using chitosan/zeolite composite from aqueous solutions: Kinetics, isotherms, and process optimization, Journal of Industrial and Engineering Chemistry 51 (2017) 185–195.

[5] I. Langmuir, The constitution and fundamental properties of solids and liquids. Part I. Solids, J Am Chem Soc 38 (1916) 2221–2295.

[6] H.M.F. Freundlich, Over the adsorption in solution, J. Phys. Chem 57 (1906) 1100–1107.

[7] S.I. Yacoub, S.G. Saber, R.A.M. Ali, E.C. Lima, G.S. dos Reis, E. Al-Olayan, Y.F. Salama, M. Mobarak, M.K. Seliem, CTAB-modified alkali-activated binder derived from Favia corals and glass waste: A novel bio-based adsorbent for effective removal of Mn(VII) ions from aqueous solutions, Journal of Industrial and Engineering Chemistry 147 (2025) 406–421.

[8] A.S. Elshimy, M. Mobarak, J.S. Ajarem, S.N. Maodaa, A. Bonilla-Petriciolet, Z. Li, M.A. Korany, D.S. Ammar, D.G. Awad, S.A. Elberbash, M.K. Seliem, Sodium alginate-modified alkali-activated eggshell/Fe3O4 nanoparticles: A magnetic bio-based spherical adsorbent for cationic dyes adsorption, Int J Biol Macromol 256 (2024) 128528.

[9] M.K. Seliem, A.S. ElShimmy, M. Mobarak, A.Q. Seliem, A.M. Salah, Z.M. Almarhoon, Z. Li, Y.F. Salama, H.I. Bendary, A magnetic bio-based adsorbent prepared from Fe3O4 nanoparticles impregnated with diatom frustules and sodium alginate for methylene blue uptake: advanced modeling and mechanism, Sep Sci Technol 59 (2024) 99–111.

[10] S.I. Yacoub, S.G. Saber, R.A.M. Ali, E.C. Lima, G.S. dos Reis, E. Al-Olayan, Y.F. Salama, M. Mobarak, M.K. Seliem, CTAB-modified alkali-activated binder derived from Favia corals and glass waste: A novel bio-based adsorbent for effective removal of Mn(VII) ions from aqueous solutions, Journal of Industrial and Engineering Chemistry 147 (2025) 406–421. https://doi.org/10.1016/J.JIEC.2024.12.031.

[11] M.A. Arif, H.A. Abdel-Gawwad, A.S. Elshimy, M.K. Seliem, M.A. Ali, S.N. Maodaa, K. Federowicz, M. Mobarak, H.I. Bendary, Y.F. Salama, M. Abd Elrahman, H. Soltan Hassan, Facile synthesis and characterization of metakaolin/carbonate waste-based geopolymer for Cr(VI) remediation: Experimental and theoretical studies, Inorganica Chim Acta 564 (2024) 121939.

[12] A.S.A.A. Abu Sharib, M. Mobarak, A.S. Elshimy, N. Al-arifi, Y.F. Salama, Z. Li, A.Q. Selim, M.K. Seliem, Facile and green fabrication of an effective and low-cost alkali-activated binder using carbonized limestone: Characterization, experiments, and statistical physics formalism for ibuprofen adsorption, Sustain Chem Pharm 41 (2024) 101701.
